# A smartphone serves as a data logger for a fully automated lab-constructed microfluidic system

**DOI:** 10.1016/j.mex.2024.102584

**Published:** 2024-01-23

**Authors:** Maitham Najim Aboud, Kamail H. Al-Sowdani

**Affiliations:** Chemistry Department, College of Education for Pure Sciences, University of Basrah, Basrah, Iraq

**Keywords:** Full automation lab-constructed microfluidic flouriometric system using a smartphone as a data logger, Automation, Microfluidic, Lab-constructed, Fluorometric system, Smartphones

## Abstract

Fluorescence is an innovative technique that has captivated scholars in recent years due to its superior sensitivity and selectivity. The development of microfluidic components has added to its appeal, particularly given the technology ability to control fluid using very small quantities (microliter range) and achieve high liquid throughput. We have combined these two technologies to develop a lab-constructed simple system for measuring fluorescence, notable for the following features:

• The device constructed entirely in our lab and programmed for measuring the fluorescence of liquids using microfluidic technology, delivered excellent results. The regression coefficient R² (0.9995) was obtained five points between 0.001-0.01µg .ml^−1^. Moreover, the reproducibility standard deviation (%) of 0.008 µg .ml^−1^ fluorescein dye remained at zero, for ten repeated experiments.
• The device was full automated using a smartphone as a data logger, and lab-constructed programs.
• The results were satisfactory with a detection limit of 1 × 10^−4^ µg.ml^−1^. This proposed system can measure over 200 samples per hour making it highly efficient and eco-friendly due to the reduced use of reagents and lower waste production. The fully automated system can effectively be used to determine fluorescein dye concentrations. Another application (micro pump view) manages all actions required in this microfluidic system, such as operating the two lab-constructed peristaltic pumps.

## Graphical abstract

**Figure:**
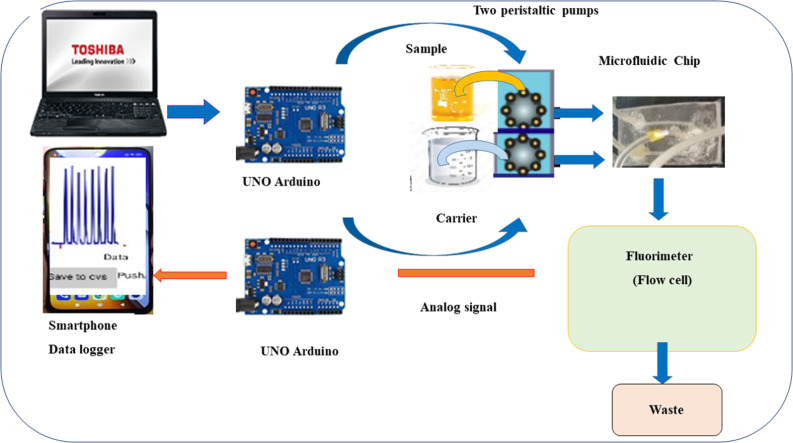

Specifications table

**Table:** 

Subject area:	Chemistry
More specific subject area:	We present the successful design of a portable smartphone-assisted radiometric fluorescence sensor employing fluorescent, which will be useful in biological and industrial applications. It can also be utilized for postgraduate research. This combination of cellphones and fluorescent test proved cost-effective and time-saving, offering an alternate technique for qualitative differentiation and semi-quantitative analysis of fluorescent dyes. Pave the way in creating clever, sensitive, and visual detection methods
Name of your method:	Full automation lab-constructed microfluidic flouriometric system using a smartphone as a data logger
Name and reference of original method:	*N.A*
Resource availability:	*ON REQUEST*

## Method details

We have designed a microfluidic device to measure fluorescein dyes. This comprehensive system contains several components allowing precise tuning of the results. It uses a smartphone and a customized program for controlling the measurements of fluorescent dyes. The system setup includes a smartphone as a controller, an UNO Arduino, a microfluidic chip, pumps, and a flow cell, all connected to a computer for results display. We gauged fluorescein dyes concentrations in micro-units using dyes as samples. The quantity of fluorescein dyes and the target application will guide the future applications of this advanced microfluidic device.

## Method validation

### Overview

Microfluidics is a scientific field that focuses on understanding small fluids system ranging from meters to nanometers in scale [1]. It leverages components like mixers, reactors, valves, and pumps to manage fluid transport processes. A development in this field, known as "lab-on-a-chip'', combined microfluidic chip with an ecosystem. This technology, which is produced industrially confers numerous advantages such as reduced sample and reagent usage and low cost-effectiveness [2,3]. Fluorescence due it superior sensitivity and selectivity, is a revolutionary method that has garnered substantial interest among researchers. Over the last few decades, microfluidics has demonstrated its significance in chemistry and biology. The field stands out for its precise fluid control employing small fluid volumes and achieving high fluid throughput [4]. Microfluidics has minimized the scale of laboratory experiments, allowing for the adjustment and operation of fluids microliter ranges. In various ways, it has proven to be an influential tool [5,6]. The influence of automation on numerous human activities has yielded significant improvements in system performance [7,8]. The complex advancements in automation technologies had a profound influence on clinical laboratories. Many manual asks in these facilities have been partially or fully replaced by automated labor-saving devices [9,10]. This paper reviews an extensive body of work centered on fluorescence technology with a particular focus on the development and creation of hand-built automated systems utilizing a fluorimeter as a detector [11]. With suitable software, smartphones have recently emerged as potential alternatives to spectroscopic instruments like spectrophotometers [12]. The availability and affordability of advanced smartphone cameras have made mobile health device design a viable option [13]. Attributes that can be incorporated into smartphones facilitate data processing and signal handling capabilities [14,15]. In this study, we utilized a smartphone outfitted with custom software. The Uno microcontroller wirelessly transmits digital signals to the smartphone via Bluetooth. Signals received were displayed as peak heights on the smartphone screen, corresponding to the concentration of the sample of interest. Our work aimed to develop a cost-effective, high throughput user-friendly, and reliable automated microfluidic system with a smartphone serving as both a data logger and detector.

### Procedure of measurements

All solutions were made using deionized distilled water. The mean value was obtained from three successive peaks heights. A 0.01 mol/L solution was prepared by dissolving 0.33231 g of fluorescein into 1 L of distilled water. A stock dye solution of fluorescein sodium salt of 0.001 mol/L was also prepared by dissolving 0.09407 g in an appropriate volume of deionized water. We ensured that the working processes were carried out on a daily basis and stored in a dark setting prevent any photo-degradation. This was achieved by covering the container carrying the stock solutions with aluminum foil. Every calibration run started with freshly prepared solutions [16], [17], [18].

### Instruments

**Fig. 1:**
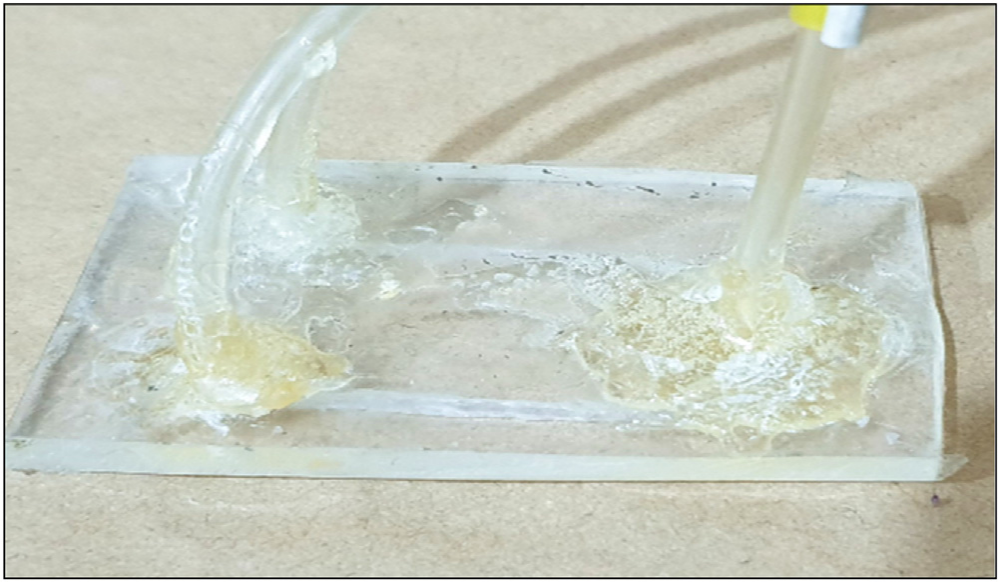
Shows the created microfluidic chip for the automated system.

The microfluidic chip used in this study was made from polydimethylsiloxane (PDMS) and built following a two-way design. The channels of the chip were shaped using a Rongxin tool, Fig. 1. We determined the volume of these channels by injecting a highly concentrated fluorescein dye with a Hamilton syringe. With each channel holding 15 µL; the chip had a total volume of 30 µL, considering the modest amounts of fluorescein sample and carrier.

### The lab-constructed mini peristaltic-pumps

**Fig. 2:**
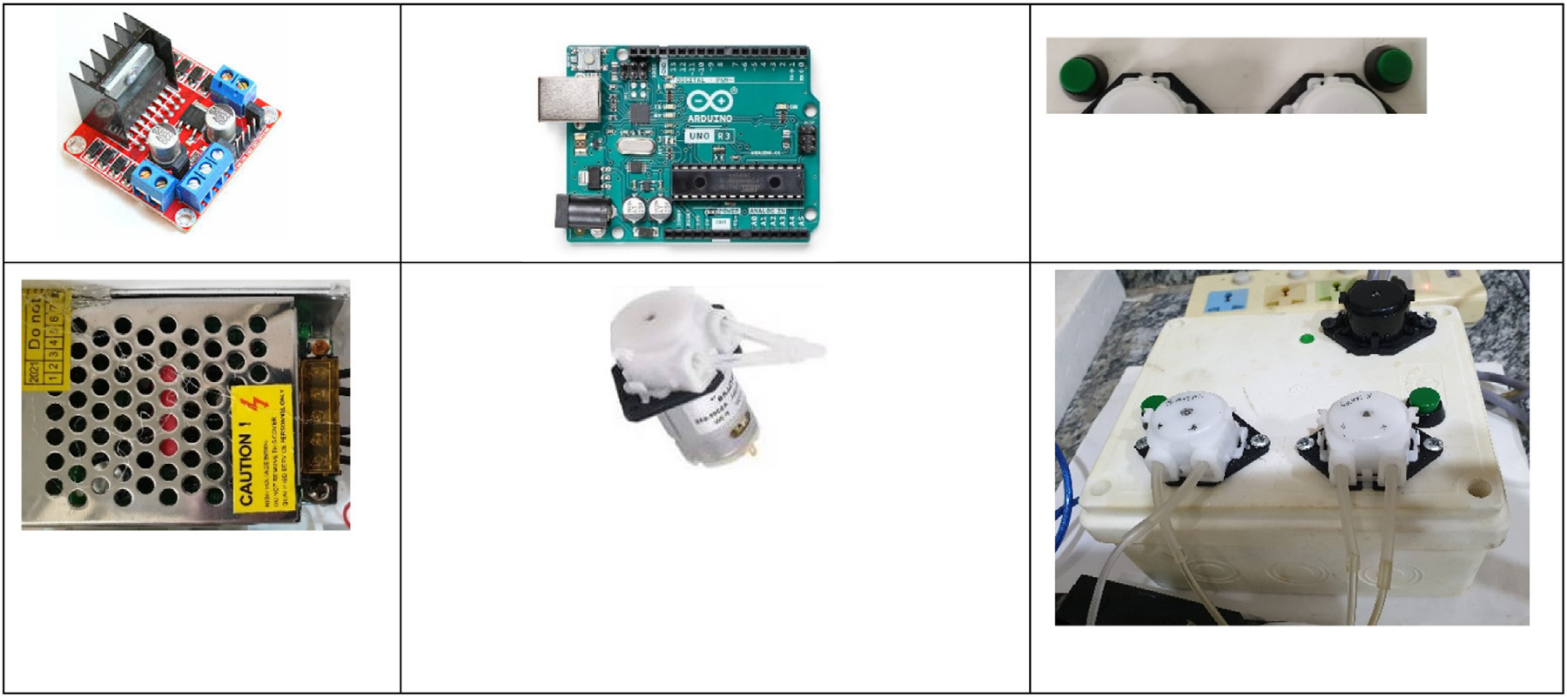
Mini peristaltic pump devices created at the laboratory.

**Fig. 3:**
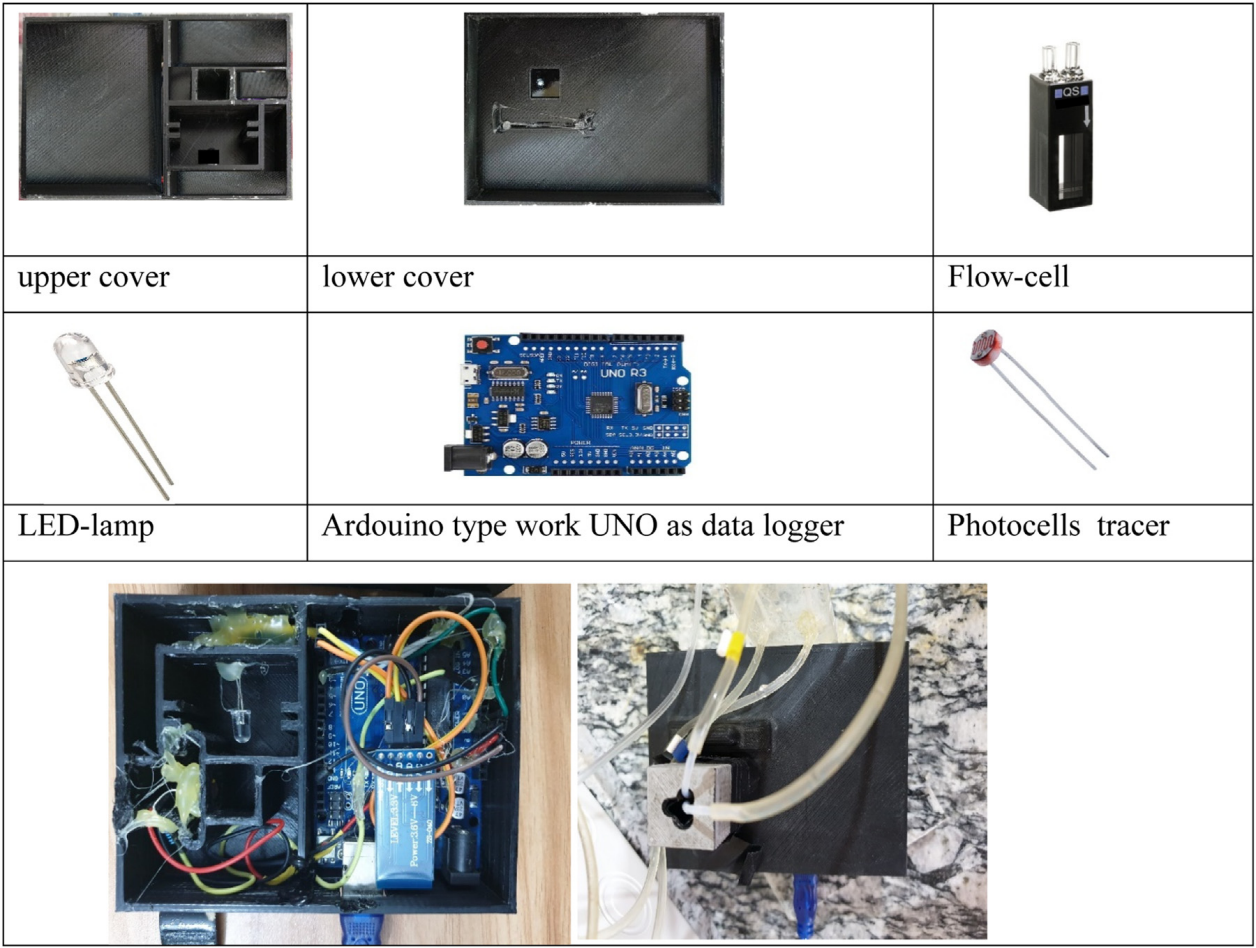
Lab-constructed Fluorometer tools.

Table 1 and Fig. 2, present the components used to construct two peristaltic-pumps. Both pumps were operated by a pair of UNO Arduino microcontrollers, with a smartphone equipped with a custom software used for data logging, Fig. 3. The signal was logged as peak height using software named Lab-Fluorometric GetData LFGD.

**Table 1:** The micro-controller components.

Component	kind	action	source
Arduino	UNO	Micro controller	Italy
Driver motor	L298N	Micro controller	China
Tow peristaltic Liquid pump dosing pump	INTLLAB 12V DC DIY	for withdrawing carrier and reagent	China
Power supply	-	Device power controlling	China
On/off button	-	Electrical power on/off	China
Two button	-	Air suction	China

### Lab-constructed fluorometer

**Fig. 4:**
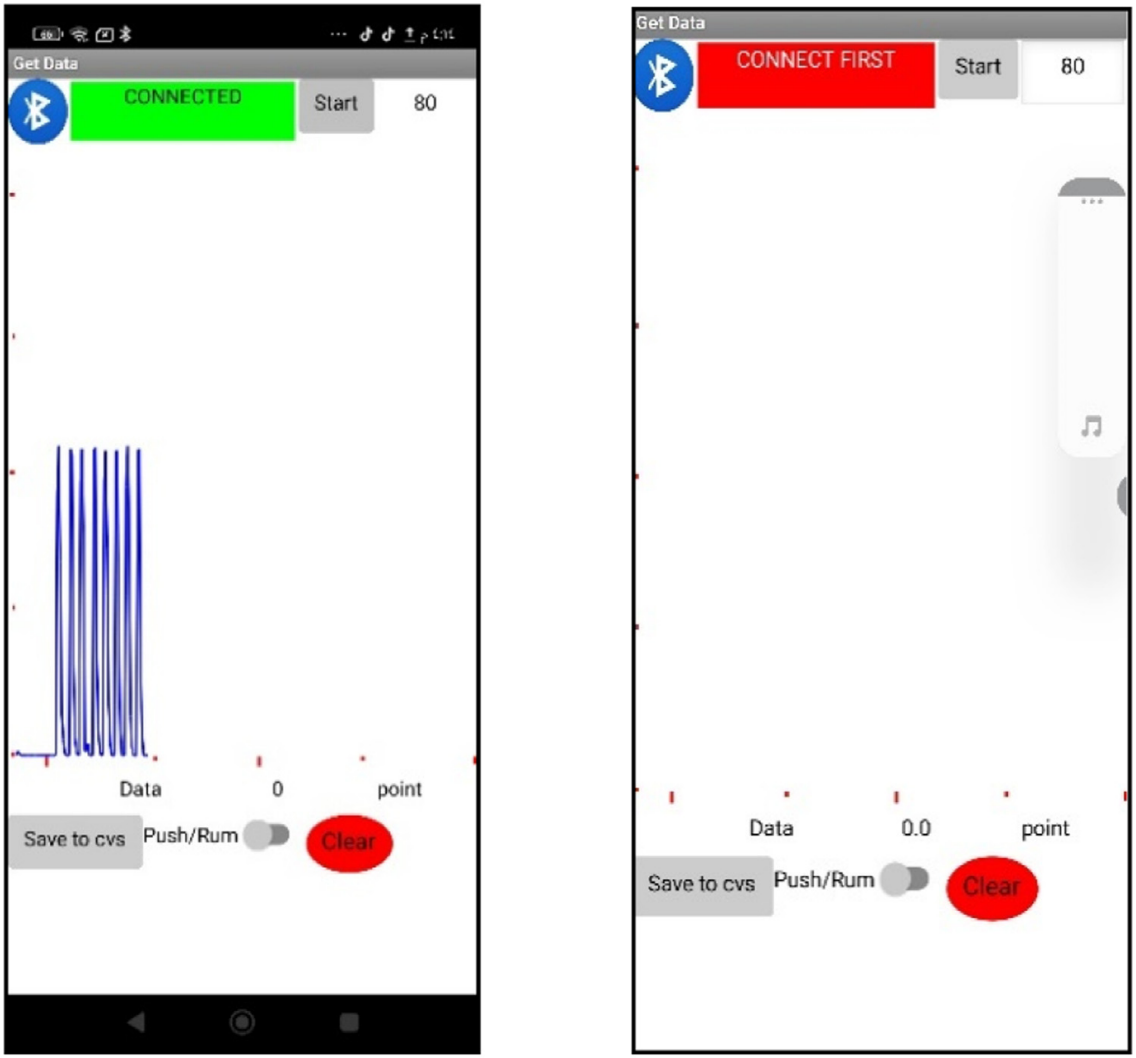
Mobile phone program view (Mi.data.florescent).

The key components of the handmade fluorometer are outlined in Table 2 and Fig. 3. A 28V LED lamb serves as a light source. The fluorometer equipped with a flow cell 450 L, and uses a photocell sensor tied to a secondary microcontroller (UNO Arduino), to detect signals. These signals are displayed as peak heights on a smartphone screen equipped with Lab-constructed software that measures the concentration of fluorescein, Fig. 4 [19], [20], [21].

**Table 2:** The lab-constructed fluorometer tools.

Tools	kind	action	source
Arduino	UNO	Micro controller	Italy
LED lamp	-	Light source	China
Photocells sensor	-	detector	China
Flow cell 450 µL	Helmma	Sample container	Germany

## Methodology

**Fig. 5:**
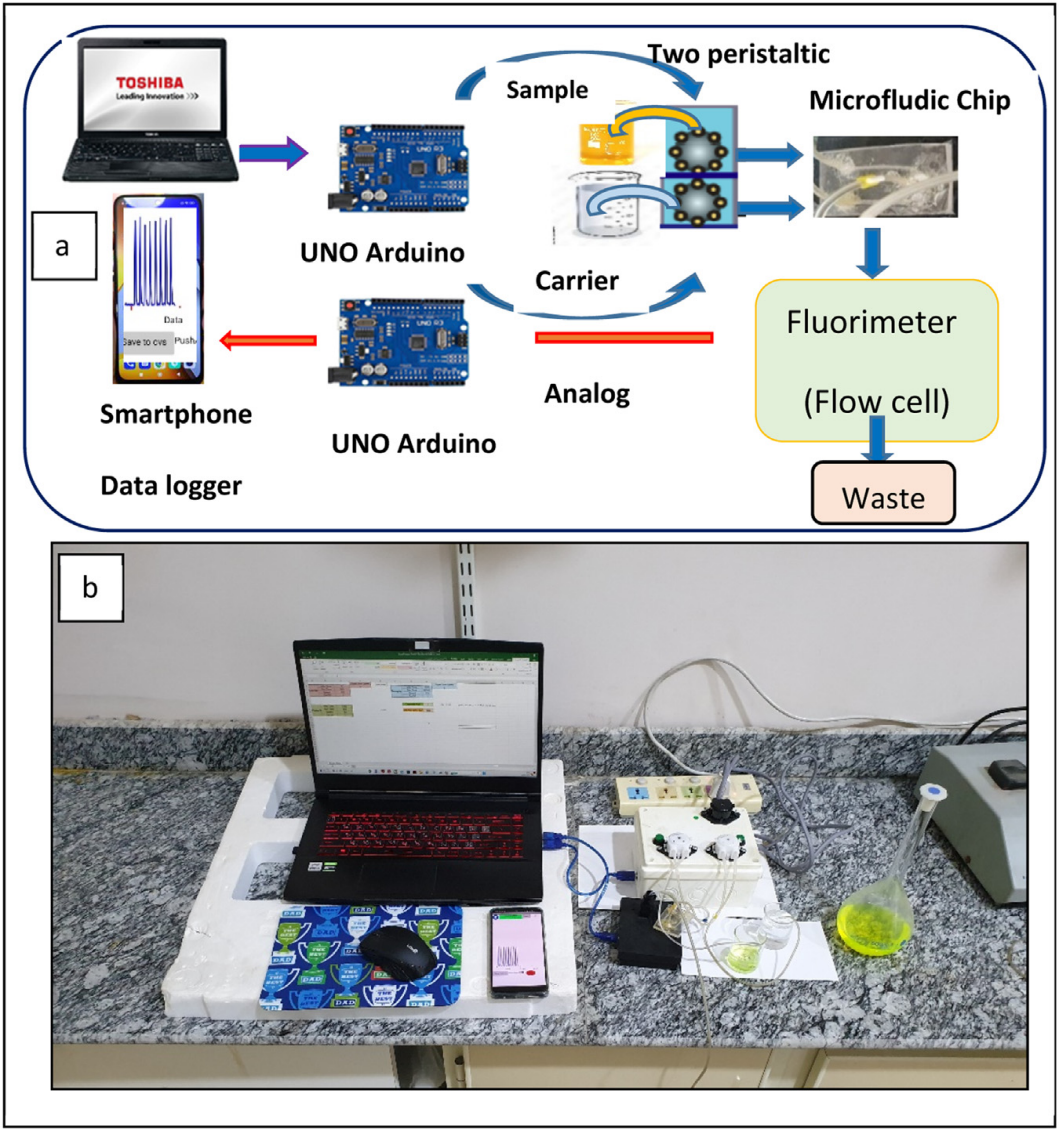
**a**- The diagram of automated microfluidic fluorometric smartphone system. **b**- The live screen of automated microfluidic fluorometric smartphone system.

Fluorescence has many practical applications, including in mineralogy, gemology, medicine, chemical sensors, fluorescent labeling, dyes, biological detectors, cosmic-ray detection, vacuum fluorescent displays, and cathode-ray tubes. The aim for future devices is to create intelligent devices with smart phones as controllers, resulting in fast, sensitive, user-friendly, and advanced control systems. These are used to characterize molecular environment and samples. To enable measurements for digital photochromic studies, adjustment may be made to the sensors of the original fluorescence device, and the smartphones software interface [22]. Our research results, shown in Fig. 5 a and b, demonstrate the automated microfluidic fluorometric smartphone system handling fluorescein dye. Automation in this system involved the use of Excel 2016 on a laptop to control the volume and timing of dye distribution via a first Arduino microcontroller. The volume of fluorescein dye was input into the first two channels of the microfluidic chip, with water subsequently added to the second channel. As the flow cell became filled with fluorescein dye, the Photocells sensor detector identified the resulting signal. A smartphone equipped with custom software recorded this signal, indicating the fluorescein concentration as a peak height.

## Results and discussion

*In situ* analysis is becoming a critical objective in analytical chemistry, aided by the rise of portable analytical devices. An ideal analysis system comprises several components that enable extraction, detection, and quantification of targeted analyses. However, preparing a portable quantification methods remain a challenge in developing such systems. Traditional lab analytical tools lack mobility, restricting their effectiveness for in depth *in situ* analysis. Smartphones, a contemporary sensation are rapidly gaining in popularity and evolving in performance. Numerous methods can be used to make transform smartphones into effective quantifiers. These include but are not limited to optical detection (spanning from colorimetric, fluorescence, chemiluminescence, bioluminescence, and photoluminescence detections to pixilation, and label-free detection), electrochemical detection, barcode reading, chemometric applications, and fluorescence microscopy with smartphone imagining. Smartphone might well represent a new direction in analytical chemistry [23].

### Optimum conditions

**Fig. 6:**
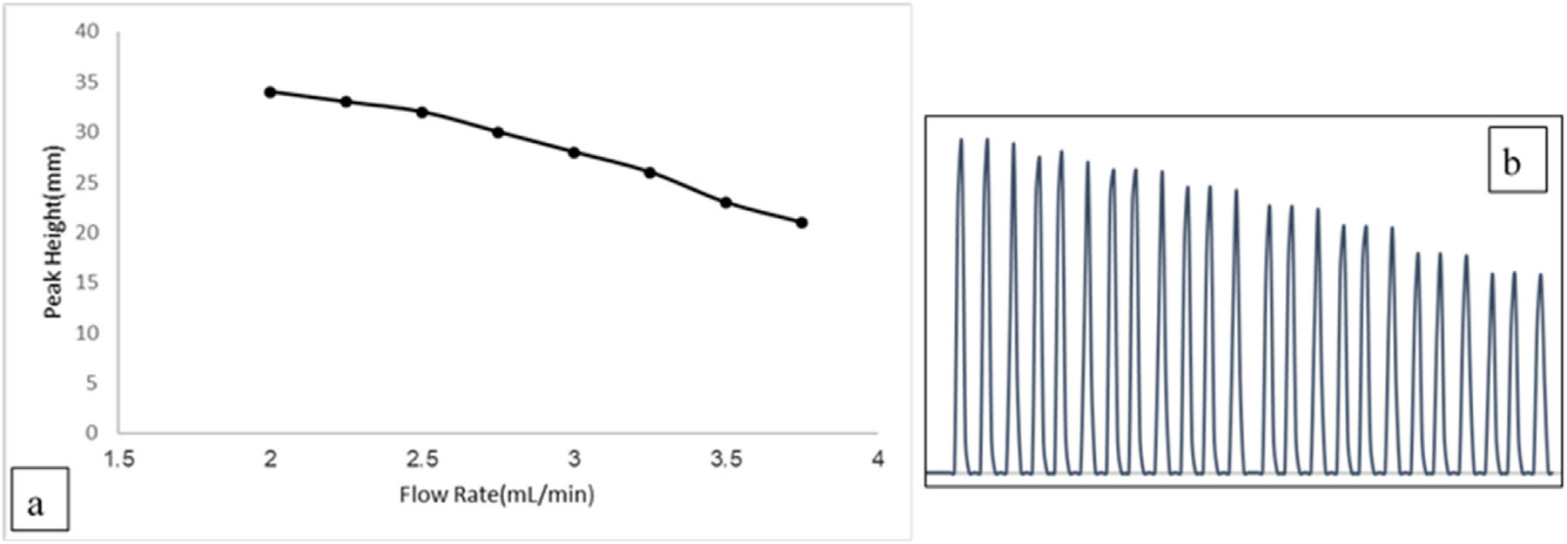
a: Flow rate effect on a peak height resultant from withdrawing fluorescein 0.008µg/ml. b: Shows the result of flow rate on the peak height of (0.008 µg/ml) from Fluorescein.

To achieve optimal performance, the measurements of fluorescein were done at 494 nm and 512 nm for excitation and fluorescence wave-lengths, respectively. Influential factors for the fully automated Lab-constructed microfluidic system were optimized. Fig. 6 and Table 3 illustrate the impact of carrier stream flow rate on the peak height of fluorescent dye (0.008 µg/mL). Our finding suggesting that an increased flow rate results in a decreased peak height, likely due to heightened fluorescein dispersion [24]. Thus, a flow rate of 2 mL/min was identified as a suitable for further investigation.

**Table 3:** List of flow rate & the peak height.

Flow rate (ml/min)	Peak height (mm)
2	34
2.25	33
2.5	32
2.75	30
3	28
3.25	26
3.5	23
3.75	21

**Fig. 7:**
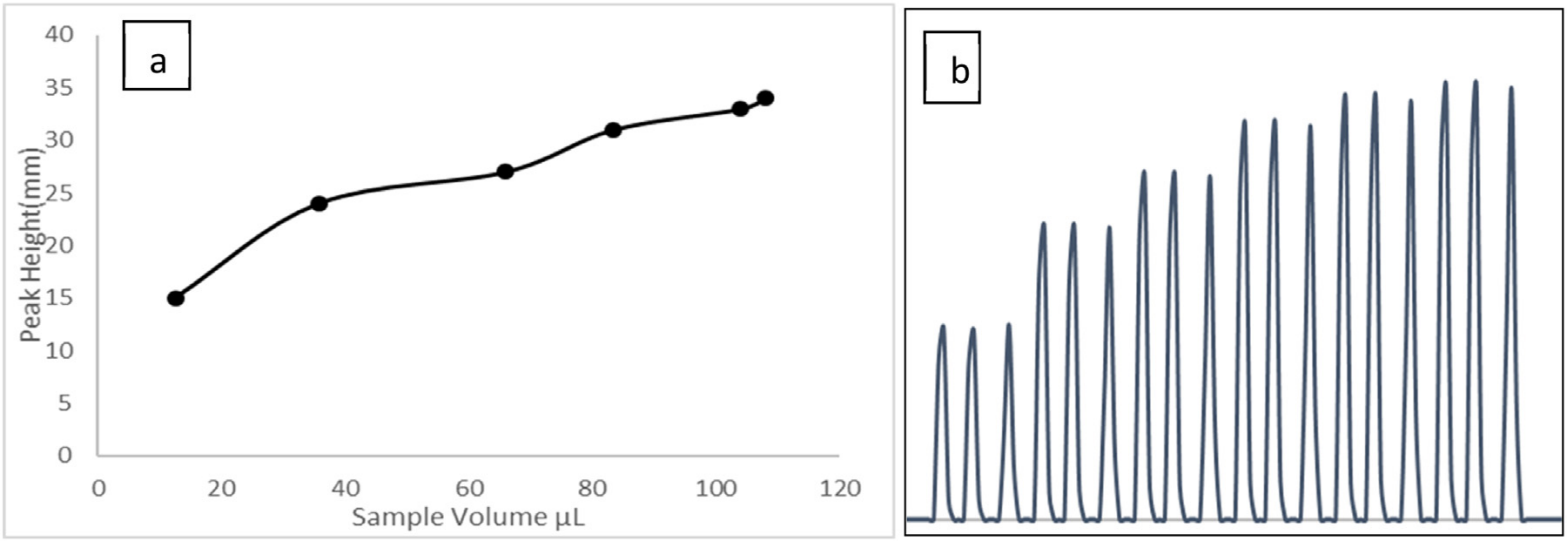
**a**: Shows the influence of the sample volume on the peaks height. **b**: The obtained peaks from system.

Fig. 7 and Table 4 demonstrate the impact of sample volume on peak height, with studied values ranging 108–12.5 µL. The results clearly indicate that peak height increases with sample volume, likely due the dispersion of greater amounts of fluorescein dye. Therefore, a sample volume of 108 µL was selected for future studies to ensure optimal sensitivity.

**Table 4:** List of sample volume and the peak height.

Sample volume (µ L)	Peak height
12.5	15
35.7	24
66	27
83.3	31
104	33
108	34

**Fig. 8:**
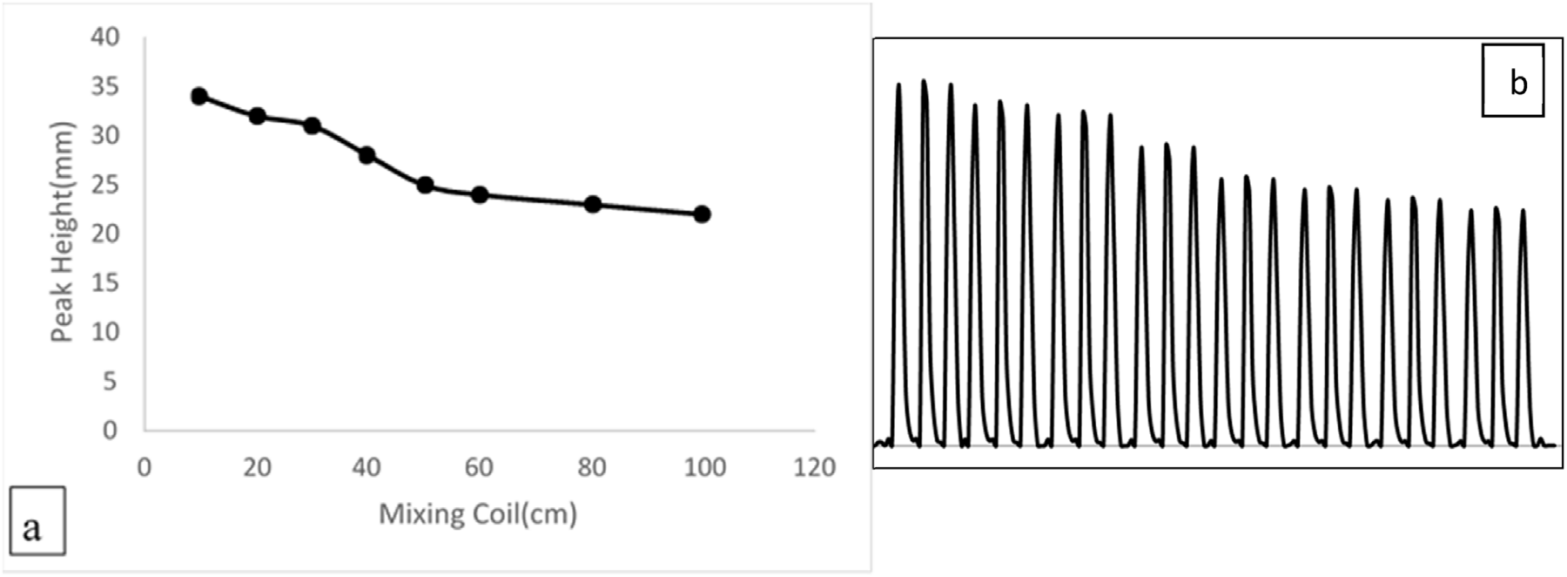
**a**: The mixing coli effect on the peak height of (0.008 µg/ml) from fluorescein. **b**: The tube length effect on the peak height

Fig. 8 and Table 5 illustrate the impact of varying coil lengths, ranging from 10 to 100 cm on the peak heights resulting from the extraction of 0.008 µg.ml^−1^ of fluorescein dye. As coil length increases, the height of the peaks reduces due to increased dilution. A coil length of 10 cm has been identified as the optimal measurement.

**Table 5:** List of tube length and the peak height.

Tube length (cm)	peak height (mm)
10	34
20	32
30	31
40	28
50	25
60	24
80	23
100	22

### Standard calibration curve

**Fig. 9:**
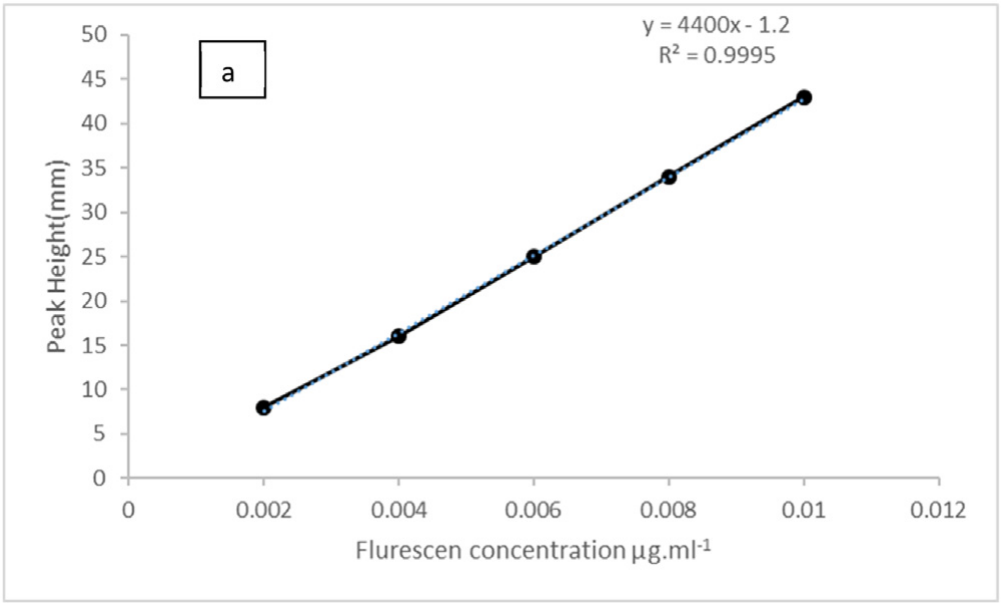
Calibration curve for fluorescein.

**Fig. 10:**
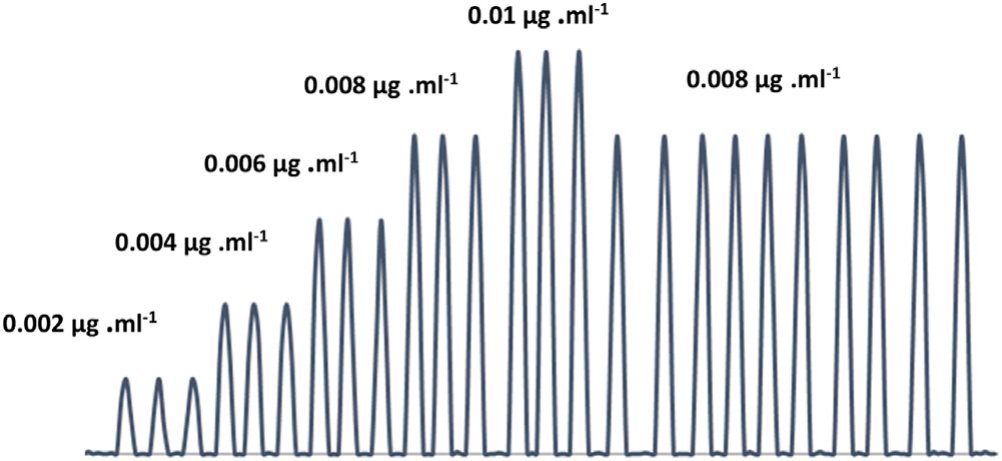
The peak height that corresponds to the fluorescein calibration curve.

Table 6 presents the obtained results. Figs. 9, 10 along with Table 7, indicate that the linearity ranged between 0.001 and 0.01 µg.ml^−1^. For the handmade system, a regression coefficient R2 of 0.9995 was noted for five points. The detection limit and the dispersion coefficient stood at 10^−4^ µg.ml^−1^ and 1.0656 respectively, Fig. 12. The system could handle 200 samples per hour, with each sample requiring 0.025mL of 0.008 µg.ml^−1^ fluorescein. Remarkably, 0.008 µg.ml^−1^ fluorescein showed zero variability across ten repetitions.

**Table 6:** optimum conditions for determination of 0.008 µg .ml^−1^ Fluorescein dye.

Parameters	Values
Total flow rate	2 ml/min
Chip volume	30 µL
Sample volume	108µL
Tube diameter	0.2 mm

**Table 7:** Standard calibration curve of fluorescein.

Fluorescein concentration (µg .ml^−1^)	Peak height(mm)	R.S.D %
0.002	8	0.0
0.004	16	0.0
0.006	25	0.0
0.008	34	0.0
0.01	43	0.0

### Recovery

**Fig. 11:**
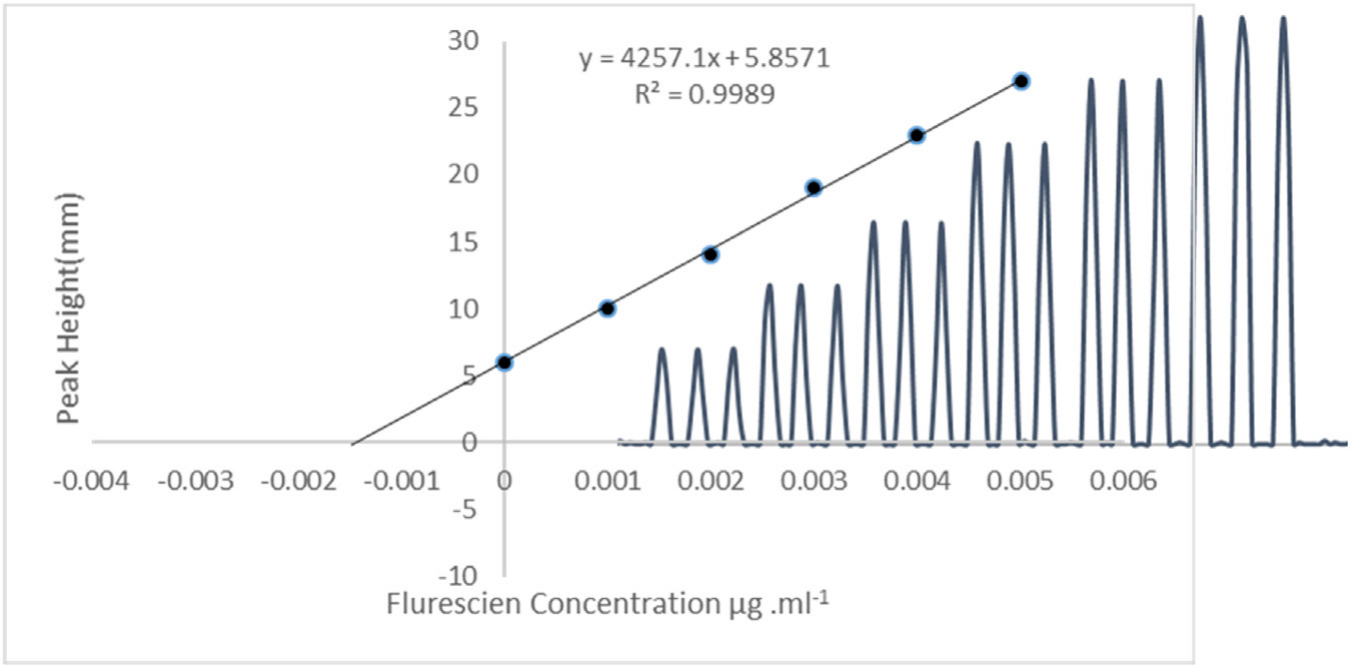
Standard addition method of adding 0.0015 µg .ml^−1^ of fluorescein sodium as a reprehensive sample (sample 1).

Table 8 and Fig. 11, present the representative sample of fluorescein sodium analyzed using the Standard Addition Method to mitigate any potential interferences. The recovery values fell within the accurate statistical range of 100–102 [25]. Three separate fluorescein sodium samples with concentrations 0.0015, 0.0025 and 0.0035 µg.ml^−1^ respectively, were individually augmented using the Standard Addition Method. Their concentrations 0.001, 0.002, 0.003, 0.004 and 0.005 µg .ml^−1^ of fluorescein, were assessed using this approach.

**Table 8:** The recoveries were investigated using the conventional adds procedure.

Sample	Added (µg .ml^−1^)	Found (µg .ml^−1^)	*Recovery
1	0.0015	0.0015	100±0.0
2	0.0025	0.0025	102±0.0
3	0.0035	0.0035	101±0.0

* RSD = zero

### Dispersion coefficient

**Fig. 12:**
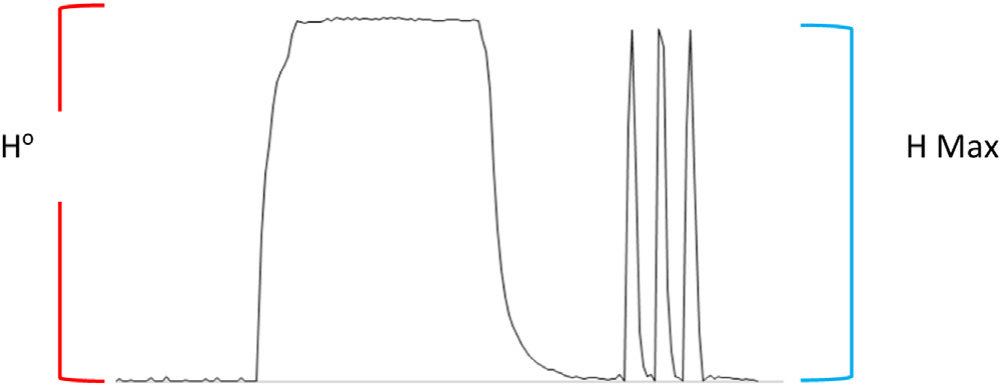
Shows the dispersion coefficient.

Fig. 12 demonstrate the calculation of dispersion coefficient in the suggested system's manifold, which was 1.051. D= H^o^/H max; D=35.76/ 34.0=1.051

### Application

We effectively utilized the proposed approach to identify representative samples of fluorescein and fluorescein sodium dye using Standard Additions Method, mitigating the impact of any interference. We confirmed the system's accuracy by using a smartphone as a data logger for this microfluidic fluorescence system, conducting data transfer over Bluetooth with high sensitivity, and processing information through custom software (Mi.data.florescent). Our Lab-on-a-chip, which conducts quantitative analysis of blood data can notably decrease analysis time, sample volume, and costs. The use of integrated sensors in combination with a smartphone facilitates direct data processing through wireless transmission for remote analysis and smartphone applications [26], [27], [28], [29], [30]. Some researchers have implemented sensor technologies for smartphone-based health monitoring. Improvements have been made in optical detection platforms, color sensors, miniaturized imaging sensors, and luminescence sensors, including photoelectric cell sensors. We can also make minor modifications to the fluorescence device to facilitate sample analysis. With the right programming, can be controlled through a smartphone interface [[31], [32], [33], [34], [35], [36]].

## Conclusions

Fluorescence spectroscopy, an emission technique uses a photon source to excite sample molecules. Molecules that emit radiation when they relax can be identified by measuring the intensity of this emitted radiation. Fluorimeter is typically used in situation when no other colorimetric method is sensitive or selective enough to identify a particualr chemical. It is widely applicable in immunoassays and bioluminescence chemistry, where it used to detect organic and inorganic substances. However, data be compromised due to various factors, such as sample contamination or interference from scattered or stray light. Therefore, it is important to always consider these potential issues. All experiments should include the collection of emission spectra and the assessment of blank samples. Smartphones equipped with quick and simultaneous quantity sensing systems are increasingly used for environmental monitoring food, safety checks, and home health care. The color changes in digital photos, detected on smartphones represent a powerful, quick, and low-cost analysis method known as digital image colorimetry. Samples can be collected with automated sampling systems, exchange systems, or repeated solution pumping all controlled wirelessly by a computer or mobile phone. A specially designed software displays the results on the smartphone screen. The approach is simple, fast, and uses minimal reagents making it environmentally friendly and fully automated, with no need for expert involvement. Moreover, it can be used in undergraduate and postgraduate laboratories for measuring graduation research, which benefits the development of their skills.

## Ethics statements

Not applicable.

## CRediT authorship contribution statement

**Maitham Najim Aboud:** Conceptualization, Methodology, Software, Data curation, Writing – original draft, Visualization, Investigation. **Kamail H. Al-Sowdani:** Supervision, Validation, Writing – review & editing.

## Data Availability

Data will be made available on request.

## Appendix Supplementary materials

Image, application 1
