# Supplementary material for: A smartphone serves as a data logger for a fully automated lab-constructed microfluidic system
Source: MethodsX. 2024 Jan 23;12:102584. doi: 10.1016/j.mex.2024.102584 (PMC10837093; doi:10.1016/j.mex.2024.102584)
Supplement: Supplementary file 1 [file mmc1.pdf]

American Manuscript Editors

English Editing Certificate

This document certifies that the manuscript listed below was edited by the expert staff of American Manuscript Editors, all of whom are native English speakers. Moreover, the document was edited for proper English language, grammar, punctuation, and spelling.

Manuscript Title:

A smartphone serves as a data logger for a fully automated lab-made microfluidic system

Authors:

Maitham Najim Aboud, *, Kamail H Al-Sowdani

Certificate Verification Key:

903-259-793-383-996

Project Number:

100094

This certificate may be verified by emailing info@americanmanuscripteditors.com. Documents receiving this certificate should be prepared for publication. However, please note that the author has the ability to accept or reject our suggestions for changes and can make changes after the editing process is complete, all of which can adversely affect the quality of the text after the editing process.

AMERICAN MANUSCRIPT EDITORS
